# Global Landscape of Human Kinase Motifs in Viral Proteomes

**DOI:** 10.1101/2025.06.02.657064

**Published:** 2025-06-03

**Authors:** Kareem Alba, Declan M. Winters, Sara K. Makanani, Prashant Kaushal, Yennifer Delgado, Immy A. Ashley, Shipra Sharma, Sophie F. Blanc, Erin Kim, Tomer Yaron, Jared L. Johnson, Justin Selby, Min Hur, Jocelyn Ni, Jasmine Nguyen, Marc H. Brent, Elaine Yip, Ahmad Kassem, James Wohlschlegel, Oliver I. Fregoso, Ting-Ting Wu, Melody M H Li, Mehdi Bouhaddou

**Affiliations:** 1 Department of Microbiology, Immunology and Molecular Genetics, University of California, Los Angeles, Los Angeles, CA, USA; 2 Institute for Quantitative and Computational Biosciences, University of California, Los Angeles, Los Angeles, CA, USA; 3 Molecular Biology Institute, University of California, Los Angeles, Los Angeles, CA, USA; 4 Department of Chemistry and Biochemistry, University of California, Los Angeles, CA, USA; 5 Columbia University Vagelos College of Physicians and Surgeons, New York, NY, USA; 6 Dana-Farber Cancer Institute, Harvard Medical School, Boston, MA, USA; 7 Department of Biological Chemistry, David Geffen School of Medicine at UCLA, Los Angeles, CA, USA; 8 Department of Molecular, Cell and Developmental Biology, University of California, Santa Cruz, Santa Cruz, CA, USA; 9 Department of Molecular and Medical Pharmacology, School of Medicine, University of California, Los Angeles, Los Angeles, CA, USA

## Abstract

The successful establishment of infection relies on an ability to sense and adapt to the host signaling state. One key mechanism of virus-host sensing is host-mediated post-translational modifications of viral proteins. While viral protein phosphorylation by host kinases is known to modulate viral functions, the global prevalence of kinase motifs across diverse viruses, and the signaling pathways they reflect, remains to be systematically explored. Here, we annotated human kinase motifs in 1,505 viral proteomes and uncovered enriched motifs in viral proteins that diverged from patterns observed in human proteins. Integration of our findings with 21,606 viral protein structures and deep mass spectrometry phosphoproteomics of infected human cells revealed that surface-accessible residues were preferentially phosphorylated and exhibited greater kinase specificity compared to buried sites. Virus-enriched motifs mapped predominantly to stress, inflammation, and cell cycle pathways—key signaling hubs dysregulated during infection that are central to the virus-host arms race—most strikingly for *Flaviviridae*, *Togaviridae*, *Herpesviridae,* and *Retroviridae* families. Temporal phosphoproteomic profiling of host kinase activity during alphavirus infection revealed dynamic patterns of stress kinase activation and viral protein phosphorylation, and the inhibition of MAP kinases reduced viral replication and phosphorylation at viral motifs with specificity for ERK and JNK kinases. Our findings suggest that viruses have evolved as biosensors of the host signaling state to optimize their life cycles, revealing new antiviral opportunities aimed at disrupting virus decision-making by manipulating host signaling cues.

## INTRODUCTION

Viruses must navigate a dynamic intracellular environment to successfully establish infection, replicate, and transmit among their hosts. It is well established that viruses evolved offensive strategies, such as viral innate immune antagonists, and hosts developed countermeasures, such as the inflammatory response, to detect and neutralize viruses.^1^ However, the extent to which viruses sense and respond to host cues in real-time, and how sensing is biochemically orchestrated, remains unclear.^2^ This capacity for rapid and selective adaptation resembles a form of molecular “decision-making” to help viruses “decide” when to replicate, assemble, or exit, playing a critical role in their fitness and ability to spread.^3^ Furthermore, uncovering how viruses sense and respond to host environments may expose critical bottlenecks in their life cycle and reveal novel targets for a new class of antivirals aimed at disrupting host cues.

Viral infection is known to provoke dramatic remodeling of host signaling via post-translational modifications (PTMs).^4^ One PTM, phosphorylation, is regulated by protein kinases and phosphatases that add and remove phosphate groups at phosphoacceptor (i.e. serine, threonine, or tyrosines) residues. Phosphorylation can act as a “switch” by generating functionally distinct proteoforms in response to cellular cues.^5^ Viral proteins are no exception; phosphorylation by host kinases can expand their functional repertoire by modulating subcellular localization, protein-protein interactions, or enzymatic activity.^6^

Previous work has implicated host kinases to play a key role in regulating viral life cycles.^7^ In HIV-1, CDK2 phosphorylates the viral protein Tat at serine 16, enhancing transcription during S-phase and linking replication to host DNA synthesis.^8^ In severe acute respiratory syndrome coronavirus 2 (SARS-CoV-2), the nucleocapsid (N) protein exists in two functionally distinct forms, one that is phosphorylated and promotes translation, and one that is unphosphorylated and drives viral assembly.^9^ In influenza virus, phosphorylation of the nonstructural protein 1 (NS1) by casein kinase 2 (CK2) enhances its binding to double-stranded RNA, allowing it to sequester RNA and block activation of host antiviral responses.^10^ Together, these examples highlight how phosphorylation allows viruses to coordinate their life cycles in conjunction with host signals.

Technological advances in mass spectrometry (MS), artificial intelligence, and genetic manipulation are enabling us to dive deeper into how phosphorylation influences protein function and cellular phenotypes. For example, data-independent acquisition mass spectrometry (DIA-MS) has enabled an unprecedented exploration into the phosphoproteome.^11^ In parallel, improvements in artificial intelligence–based structural modeling using AlphaFold^12^ have made it possible to accurately predict and interrogate viral protein structures.^13^ Furthermore, recent advances in kinase motif prediction, such as the Kinase Library^14^, which integrates positional scanning peptide array data with computational prediction tools, allow researchers to assess kinase specificity for any given protein sequence. The integration of these technologies support the investigation of host kinase-mediated phosphorylation of viral proteins by enabling both large-scale detection by MS, structural interrogation by AlphaFold, and sequence-based kinase motif prediction by the Kinase Library.

In this study, we harnessed these tools to perform a global analysis of human kinase motifs across thousands of viral proteomes. By integrating kinase motif predictions for viral proteins, phosphoproteomics of infected cells, and viral protein structural modeling, we identified conserved signaling pathways that viruses appear to sense on the surface of their proteins across diverse families. We further validated the functional significance of this sensing strategy by perturbing MAPK signaling during Sindbis virus (SINV) infection, revealing defects in both viral protein phosphorylation and replication. Together, our findings illuminate a previously underappreciated layer of viral-host interaction in which human kinase activity is co-opted, not just defensively by the host, but offensively by viruses to interpret and manipulate their environment.

## RESULTS

### A diverse array of viruses encode high confidence human kinase motifs

**Figure 1.**
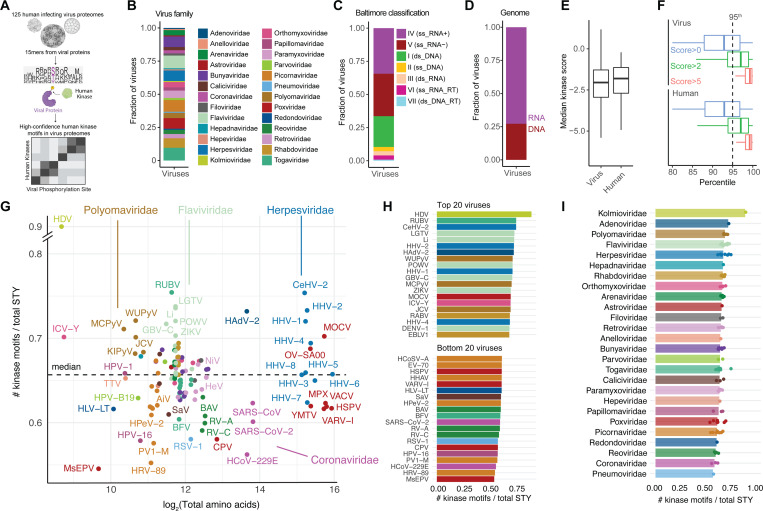
Diversity of kinase motifs present in viral proteomes. (A) A total of 125 viral proteomes were collected, and kinase specificity for various motifs was predicted using the Kinase Library algorithm. (B) The distribution of viral families represented in the dataset. (C) The distribution of Baltimore classifications among the viral proteomes. (D) The distribution of genomic material types present in the dataset. (E) A comparison of the median kinase motif scores between viral and human proteomes. (F) The distribution of kinase motif scores across viral and human proteomes. (G) The relationship between the log_2_-transformed proteome size and the number of high-confidence motifs, normalized to the number of phosphoacceptor sites. (H) The top 20 and bottom 20 viruses ranked by the number of kinase motifs normalized to phosphoacceptor count. (I) The number of kinase motifs per phosphoacceptor site grouped by viral family.

To investigate the capacity for viral proteins to possess human kinase motifs, we assembled 125 proteomes from human-infecting viruses and subjected each phosphorylation acceptor residue (i.e. serine, threonine, or tyrosine), and the surrounding seven upstream and downstream amino acids, referred to as a “peptide”, to computational kinase motif analysis (Table S1). Specifically, we employed The Kinase Library^14^ algorithm, a recently developed algorithm informed by positional scanning peptide array (PSPA) analysis (Fig. 1A). The Kinase Library provides a score for every site-kinase pair; a score of 0 indicates a random association, a positive score indicates a favorable interaction, and a negative score indicates an unfavorable interaction (i.e. negative selection). The 125 viruses spanned 26 viral families (Fig. 1B), all seven Baltimore classifications (Fig. 1C), with a slight bias for RNA versus DNA-based genomes (Fig. 1D). Viral and human proteins depicted equivalent median kinase scores, which trended negative, thought to indicate a trend towards negative selection away from phosphorylation for both organisms (Fig. 1E) consistent with prior findings.^14^ A percentile score was used to compare kinase favorability for each peptide relative to 80,000 known human phosphorylation sites. We found the positive kinase score distributions to be similar in terms of their percentile ranking across viral and human proteins and defined a high-confidence kinase motif as possessing a score greater than 2, which we found exceeds the 95% percentile (Fig. 1F).

We then ranked viruses by the number of high confidence kinase motifs (i.e. score > 2) divided by the number of phosphoacceptor residues (i.e. S, T, or Y) in their proteomes. We found most viruses varied between approximately 0.55 to 0.75, with a median of 0.66, which remained consistent for a variety of proteome sizes (Fig. 1G). Strikingly, we found Hepatitis D virus (i.e. delta hepatitis) to have the highest proportion (0.89) of high-confidence motifs for GSK3A, PIM1, and HIPK4 kinases, suggesting a role for these kinases in regulating its life cycle. In general, viruses in the herpesviridae, flaviviridae, and polyomaviridae, each representing 1–12 viruses, had the highest proportion of high-confidence motifs, suggesting these viruses may have evolved to sense and respond to phosphorylation signaling cascades on many of their proteins (Fig. 1G–I). Conversely, some viruses possessed a low proportion of high-confidence motifs, such as the coronaviridae, reoviridae, and picornaviridae families (Fig. 1G–I). Importantly, just because a virus has a low number of high-confidence motifs, they may still possess highly-functional phosphorylation events that serve important regulatory roles during their life cycles.

### Viral proteins encode human kinase motifs for stress, inflammation, and cell cycle pathways

**Figure 2.**
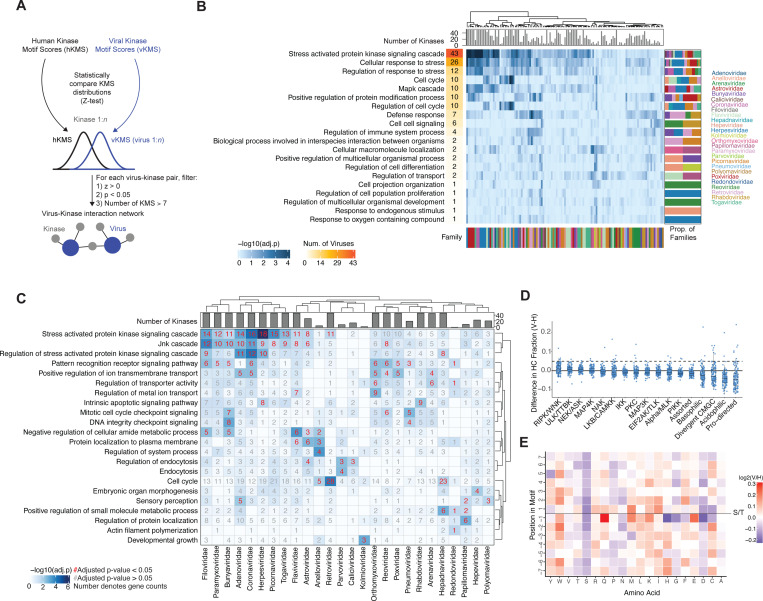
Viral kinase motifs are largely responsive to stress and cell-cycle pathways. (A) Viral kinase motif scores were compared per virus to the global human kinase motif score distribution for each kinase, generating a z-score and associated p-value. The resulting network was thresholded based on the following criteria: (1) z > 0, (2) p < 0.05, and (3) more than seven high-confidence kinase motif scores (KMS;score > 2) per virus–kinase interaction. (B) GSOA results for each virus from the filtered network, sorted by the number of significant viruses per term. (C) GSOA results performed per viral family. (D) For each virus, the proportion of high-confidence motifs for each kinase family was subtracted by the corresponding proportion in humans. (E) Enrichment of each amino acid at each position in high-confidence S/T kinase motifs compared to the base distribution in the respective proteome, calculated as the ratio between viral and human distributions.

To probe the kinase signaling pathways that were implicated in phosphorylating viral proteins, we first developed a statistical pipeline to compare kinase motif scores (KMS) between virus and human, designed to identify kinases with an enriched preference for viral relative to human substrates (Fig. 2A). Specifically, for each kinase, we performed a Z-test to compare the KMS distribution for each virus compared to all human phospho-acceptor peptides, resulting in a z-score and p-value for each virus-kinase pair. A positive virus-kinase z-score indicates that a kinase preferentially targets a given viral proteome, whereas a negative z-score suggests reduced targeting, potentially reflecting selection against phosphorylation. Next, we constructed a high-confidence virus-kinase interaction network, representing an undirected network linking each virus to associated human kinases. For each virus-kinase pair, we applied three criteria: a z-score greater than zero (z > 0), a p-value less than 0.05 (p < 0.05), and a minimum of seven high-confidence kinase motifs (high-confidence defined as KMS > 2; Fig. S1A) detected within each viral proteome (Fig. 2A; Table S2). A threshold of seven high-confidence motifs was chosen because it yielded the highest number of enriched Gene Ontology Biological Process terms across all viruses, suggesting this threshold optimally balances the sensitivity for statistical enrichment (Fig. S1A).

After identifying high-confidence virus-kinase interactions, we performed gene set overrepresentation analysis (GSOA) for each virus, using either the top 50 kinases or all available kinases if fewer than 50, to uncover the biological pathways associated with the corresponding human kinases (Table S3). The top 20 most commonly enriched terms across viruses included stress signaling cascades, MAP kinase signaling, inflammatory responses, cell cycle, protein localization, cellular differentiation, and metabolism (Fig. 2B). Interestingly, across all viruses, the most enriched pathways involved stress, inflammation, and cell cycle.

We then grouped viruses by viral family and repeated the GSOA, which revealed similar pathways as the virus-specific analysis, including stress, inflammation, cell cycle, development, and protein trafficking, among others (Fig. 2C). Herpesviridae was the most strongly enriched for the “stress activated protein kinase signaling cascade” term, which is interesting given that herpesviruses are known to respond to stress kinase signals by reactivating from latency;^15^ however, direct host stress kinase phosphorylation of herpesviruses proteins remains poorly studied. Viruses in the Coronaviridae, Picornaviridae, Adenoviridae, Filoviridae, Togaviridae, Bunyaviridae, Paramyxoviridae, Flaviviridae, Retroviridae, and Astroviridae families were also significantly enriched for stress-kinase motifs, suggesting these viruses have evolved to sense and respond to stress signals during their life cycle. For example, bovine parainfluenza virus in the Paramyxovirdiae family is known to co-opt the host p38 MAPK signaling pathway to enhance expression of viral proteins and promote efficient replication.^16^

We also found viruses in the Retroviridae and Hepadnaviridae families to be enriched for cell cycle-related kinases, which is intriguing given these viruses are known to replicate in the nucleus. For instance, retroviruses reverse-transcribe their RNA genomes into DNA and integrate them into the host genome as part of their replication cycle, known as a provirus, promoting viral persistence in the host. Integration is coordinated alongside the cell cycle stage, occurring primarily in late G1 or early S phase, and several cell cycle-related kinases have been previously implicated in the retrovirus life cycle.^17^ Hepatitis B virus (HBV), a Hapadnaviridae member, is known to prefer non-dividing cells arrested in G0/G1 or early S, when the nuclear membrane remains intact, to protect its genome and support replication. In fact, the HBV HBx protein helps maintain cells in G0/G1 or early S phase by modulating host cell cycle regulators.^18^ The extent to which HBV coordinates its life cycle alongside host cell cycle signals remains largely unexplored. Lastly, we found the Orthomyxoviridae family, which includes influenza viruses among others, to be enriched for ion transport-related kinases, which is intriguing given the known reliance of influenza on its viral M2 proton channel to acidify the viral interior during entry and to regulate pH during viral assembly and release.^19^

We next investigated whether virus-kinase interactions were enriched for specific amino acid motif categories relative to humans. To assess this, we compared the fraction of high-confidence kinase motifs (KMS > 2) assigned to each motif category, as defined by Johnson et al.^14^, and used the difference between viral and human distributions as a metric of selection for specific motif families (Fig. 2D). Overall, we found viral proteins did not possess strong selection for particular motif families relative to human proteins. However, there was notable variability between viruses, with some viruses showing clear preferences for specific motif classes. The most frequently selected motif family across viruses was RIPK/WNK.

To investigate position-specific effects across high-confidence motifs for serine/threonine kinases, we examined the frequency of each amino acid at every position within the motif, normalized to the background distribution in the base proteome (Fig. S1B–D). We then calculated the difference in normalized ratios between high-confidence viral and human motifs at each position (Fig. 2E). Overall, we observed a depletion of serines across all positions flanking viral phosphosites. In contrast, glutamine showed strong enrichment at the −1 position. Most of the observed selection, whether positive or negative, was concentrated at the −1 site directly adjacent to the phosphosite.

### Viral protein phosphorylation sites are mostly, but not always, surface exposed.

We next investigated the relationship between viral protein phosphorylation and the structural context of where the sites occurred. To do so, we integrated mass spectrometry phosphoproteomics data from virus-infected cells, viral protein structural data, and human kinase motif scores in viral proteins. We confirmed phosphorylation of viral proteins from three viruses using global MS phosphoproteomics during Sindbis virus (SINV) infection in HEK293T cells (MOI of 1), KSHV latency reactivation in BC-3G B-cells, and HIV-1 latency reactivation in JLat 10.6 T-cells (Fig. S2A and S2B). Reactivation was achieved by stimulating cells with 5 ug/mL of phorbol 12-myristate 13-acetate (PMA). Throughout our analysis, we compared all observed trends to phosphorylation data from human proteins obtained in the same experiments.

**Figure 3.**
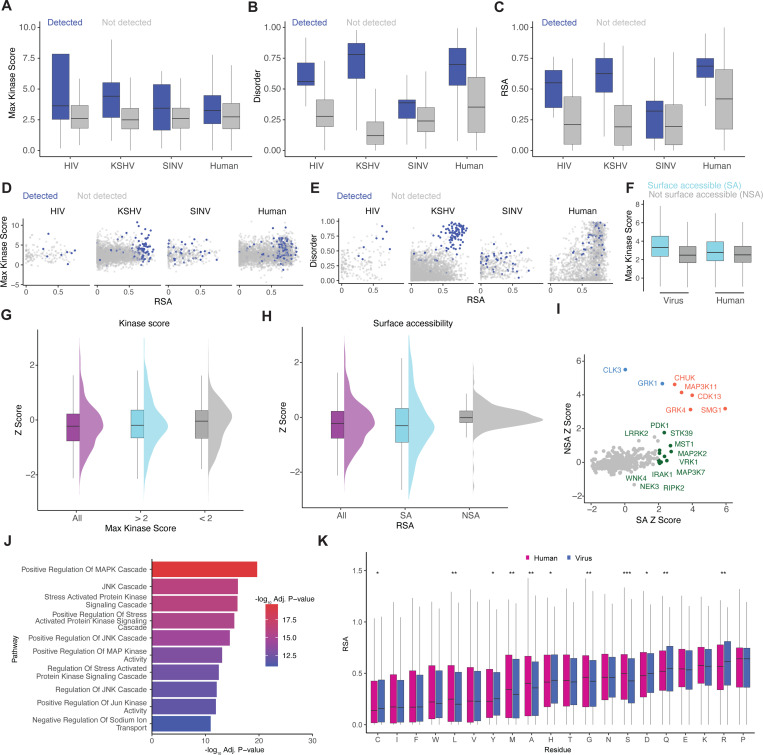
Viral proteins are responsive to phosphorylation largely on their surface. (A) Comparison of the maximum kinase motif score per site between phosphoproteomics-detected and undetected sites across HIV, KSHV, SINV, and human proteins. (B) Comparison of intrinsic disorder per site between detected and undetected sites. (C) Comparison of relative surface accessibility (RSA) per site between detected and undetected sites. (D) Relationship between RSA and maximum kinase motif score across all sites and data sources, colored by detection in phosphoproteomics data. (E) Relationship between RSA and intrinsic disorder across sites and data sources, colored by detection in phosphoproteomics data. (F) Comparison of maximum kinase motif scores per site between viral and human proteomes, colored by surface accessibility. Surface accessible (SA) sites are defined as RSA > 0.25; non-surface accessible (NSA) sites are defined as RSA < 0.25. (G) Z-scores calculated per virus–kinase pair, thresholded by whether the maximum kinase motif score was greater or less than 2. (H) Z-scores calculated per virus–kinase pair, thresholded by RSA value above or below 0.25 (SA and NSA, respectively). (I) Comparison of median Z-scores per kinase when calculated for surface-accessible versus non-surface-accessible sites. Green indicates kinases enriched only in SA sites, blue indicates those enriched only in NSA sites, and orange indicates enrichment in both. (J) Top GSOA term enrichment results from Z-scores derived from surface-accessible motifs. (K) Comparison of RSA distributions across viral and human proteomes, stratified by amino acid residue type.

Sites detected during infection exhibited higher maximum kinase motif scores (Fig. 3A) and were more likely to occur in intrinsically disordered regions (Fig. 3B). These sites also tended to be more surface exposed, as indicated by higher relative solvent accessibility (RSA) values (Fig. 3C). However, the magnitude of these trends varied across viruses: while KSHV phosphosites were overwhelmingly disordered and surface accessible, SINV phosphosites showed minimal differences between detected and undetected sites.

We next assessed the relationship between kinase motif scores, disorder, RSA, and phosphorylation. We observed a trend of increasing kinase motif scores associated with surface-exposed phosphorylation sites (Fig. 3D). We also found most detected sites on KSHV and HIV proteins to be on disordered and surface exposed residues (Fig. 3E). Surprisingly, SINV phosphosites were generally more ordered and buried within the protein core, which was also found to be the case for many human sites (Fig. 3E). This may suggest that these sites are phosphorylated during or prior to protein folding. Interestingly, we noticed the proportion of phosphorylated phospho-acceptors was much higher for SINV (10.5%) than for KSHV (3.3%), HIV (4.6%), or human proteins (5.0%), despite all having equivalent fraction of high-confidence kinase motifs (Fig. XYZ). The high fraction of phosphorylation sites in SINV may reflect their location in non–surface-accessible regions, limiting access by host phosphatases and suggesting a potential role in maintaining viral protein structural integrity.

### Human kinase specificity for viral proteins is enhanced at surface-exposed regions

To more deeply investigate the structural constraints of viral protein phosphorylation by human kinases, we integrated a previously published dataset^20^ of 1,391 viruses and 21,606 Alphafold2-generated viral protein structures with human kinase motif scores. We observed that phosphorylation sites on the surfaces (RSA > 0.5) of both viral and human proteins exhibited higher max KMS than those located in buried regions (RSA < 0.5; Fig. 3F).

Using the z-score-based approach outlined above (Fig. 2A), we next examined viral preference for specific human kinases under various filtering criteria. When filtering by max kinase score, we saw little-to-no gain in the differences between motifs on human or viral proteins (Fig. 3G; Fig. S2C, top). However, filtering by RSA revealed a clearer distinction in kinase specificity, as surface-accessible viral phosphorylation sites showed a greater divergence from their human counterparts, with z-scores displaying more variability than those of buried sites (Fig. 3H; Fig. S2C, bottom). This suggests that the surfaces of viral proteins were under greater selective pressure to favor specific kinases. Among the kinases with the highest median z-scores across viruses for surface-exposed sites were RIPK2, IRAK1, and MAP3K7, all known for roles in modulating the stress response (Fig. 3I). To globally interrogate this trend, we performed GSOA on kinases with statistically significant positive SA z-scores (p < 0.05), which confirmed enrichment for stress signaling pathways (Table S3; Fig. 3J). Since many stress kinases are known to phosphorylate motifs with a proline in the +1 position, we next systematically evaluated whether amino acid composition could account for the elevated z-scores. To do this, we compared RSA scores between humans and viruses for each amino acid and found no significant difference in the surface exposure of proline residues (Fig. 3K). However, we observed a statistically significant preference for R, Q, D, H, Y, and C on surface-exposed motifs of viral proteins, and for L, M, A, G, and S on surface-exposed motifs of human proteins (Fig. 3K).

### Dynamic regulation of Sindbis virus phosphorylation and MAPK signaling reveals a critical dependency

**Figure 4.**
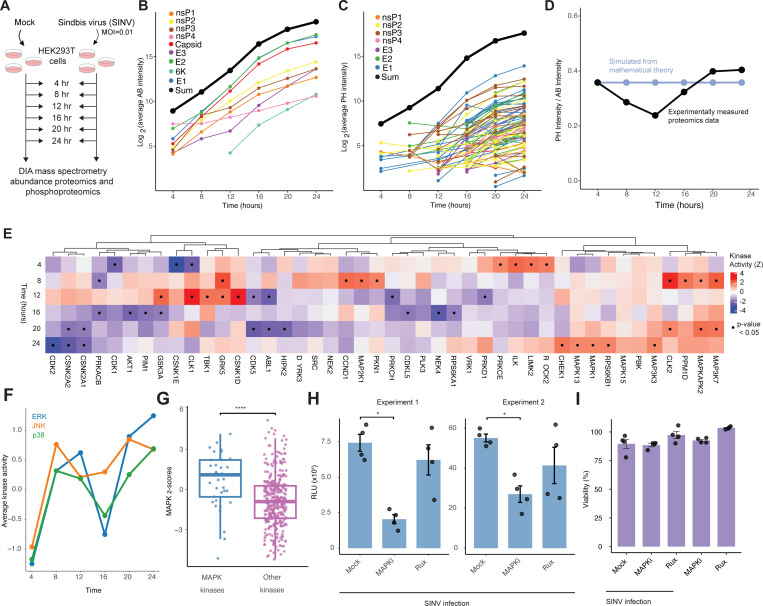
Time-course proteomic profiling and functional validation of host kinase responses during SINV infection. (A) HEK293T cells were infected with SINV (strain TE5’2J/GFP) at an MOI of 0.01, cell lysates were collected at 4 hour time intervals for DIA mass spectrometry abundance and phosphoproteomics. (B) Change in viral protein abundance over time, averaged across biological replicates. (C) Change in phosphorylation levels on viral proteins over time, averaged across biological replicates. Each line represents a different phosphopeptide. (D) Ratio of phosphorylation intensity to protein abundance over time (black), compared to model-predicted values. (E) Changes in host kinase activity over the time course compared to mock-infected controls. (F) Average kinase activity over time for kinases within the ERK, JNK, and p38 pathways. (G) Z-scores calculated for MAPK kinases in Sindbis virus compared to all other kinases (Wilcoxon rank-sum test, p < 0.0001). (H) Luciferase assay results from SINV-infected cells pre-treated with 1 uM MAPK inhibitors or 1 uM ruxolitinib one hour before infection, repeated across two independent experiments (Wilcoxon rank-sum test, p < 0.05). Cells were harvested at 24 hours post-infection. (I) Cell viability for each treatment group; 100% represents non-infected, untreated mock controls.

We next sought to probe the impact of human kinase phosphorylation of viral proteins on viral replication. We selected SINV as a model system due to its ease of use in the laboratory and its similarity to more pathogenic alphaviruses.^21^ We first performed MS abundance proteomics and phosphoproteomics of infected HEK293T cells over time (4, 8, 12, 16, 20, and 24 hours post infection) to characterize the dynamics of host kinase signaling activities and viral protein phosphorylation trends over time (Table S4; Fig. 4A). As expected, our data showed a steady increase in viral protein abundance (Fig. 4B) and phosphorylation (Fig. 4C) across the time course. Interestingly, all nine viral proteins were detected in the abundance proteomics data and phosphorylation was detected on all except the 6K protein (Fig. 4B and 4C; Fig. S3A and S3B).

We next investigated the ratio of phosphorylation to protein abundance in viral proteins to determine whether phosphorylation was enriched at specific time points. To establish a baseline, we compared this ratio to an expected distribution generated by a simulated mathematical model of enzymatic activity, which predicted a constant phosphorylation-to-abundance ratio over time (Fig. 4D). In contrast, we observed that the actual ratio increased over time, suggesting that additional factors may be contributing to this trend.

To assess the impact of infection on host signaling, we estimated host kinase activities over time from the global phosphoproteomics data using prior knowledge networks of kinase-substrate interactions, monitoring a consistent set of phosphorylated peptides for each kinase over time (Table S5; Fig. 4E). At 4 hours post infection (hpi), we noted high ROCK2 and LIMK2 activity, both cytoskeleton regulators, possibly reflecting changes that occur during viral entry. Interestingly, at 8 and 12 hours, we noted activation of MAPK and TBK1 stress and innate immune signaling, which was blunted by 16 hpi but resurged by 24 hpi (Fig. 4E). To further explore this observation, we examined the mean activity of MAP kinases over time, grouped by the JNK, ERK, and p38 subfamilies. This analysis confirmed dynamic activity patterns, with peaks observed at 8 and 24 hours (Fig. 4F). To further explore this, we examined whether sites in the SINV proteome showed stronger selection for MAPK-mediated phosphorylation. Using the Z scores previously generated for a panel of MAPK kinases, we found that SINV sites had significantly higher Z scores compared to sites associated with other kinases, indicating potential selective pressure for phosphorylation by MAPKs (p < 0.0001; Fig. 4G; Fig. S3C).

We tested the functional role of MAPK signaling in infection by using a SINV virus containing a genomic promoter driving luciferase expression.^22^ HEK293T cells were pretreated for one hour with either a control, 1 μM MAPK inhibitor cocktail (doramapimod [p38 inhibitor], JNK-IN-8 [JNK inhibitor], and U-0126 [MEK/ERK inhibitor]), or 1 μM ruxolitinib (a JAK/STAT pathway inhibitor), followed by infection with SINV strain Toto1101/Luc at a multiplicity of infection (MOI) of 1. Cells were harvested 24 hours post-infection. Across two independent experiments, we observed MAPK inhibition significantly reduced SINV genome replication, which was not observed for ruxolitinib-treated cells (Fig. 4H), an effect that could not be explained by differences in cell viability (Fig. 4I).

### MAPK inhibition reduced phosphorylation of Sindbis proteins with ERK and JNK motifs

**Figure 5.**
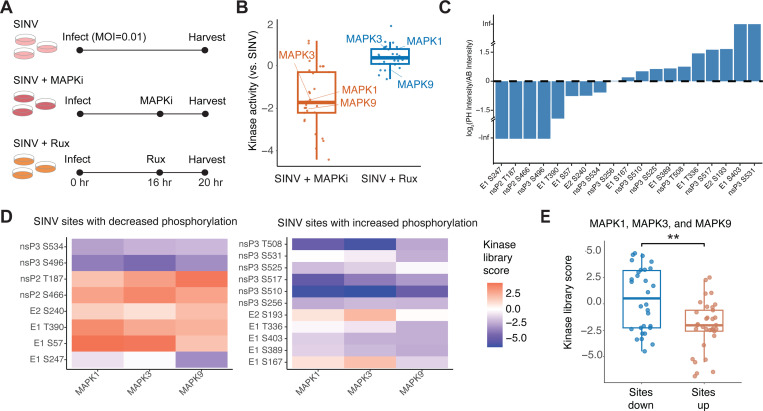
MAPK signaling regulates phosphorylation of viral proteins during Sindbis infection. (A) HEK293T cells were infected with SINV (strain Toto 1101) at an MOI of 0.01. A MAPK inhibitor cocktail or ruxolitinib was added at 16 hours post-infection, and cells were harvested at 20 hours for proteomic and phosphoproteomic analysis. (B) Kinase activity compared across SINV-infected cells with or without MAPK inhibitor or ruxolitinib treatment; contrasts are shown relative to untreated SINV infection. (C) Detection of phosphorylation sites in MAPK inhibitor-treated versus mock-treated cells, normalized to protein abundance in each condition; values greater than 1 indicate enrichment in MAPK inhibitor-treated cells. (D) Kinase Library scores for sites with increased or decreased phosphorylation, calculated for MAPK1, MAPK3, and MAPK9. (E) Comparison of Kinase Library scores for MAPK1, 3, and 9 across sites with differential phosphorylation (Wilcoxon rank-sum test, p < 0.01).

We validated this finding by infecting cells with SINV and adding either MAPK inhibitors or ruxolitinib at 16 hours post infection for 4 hours (Table S5). At 20 hours post infection, we collected cell lysates for abundance and phosphoproteomic analysis (Fig. 5A; Fig. S3D). As expected, kinase activity analysis confirmed MAPK inhibitors decreased MAPK activity relative to untreated SINV infection (Fig. 5B). Nineteen (19) viral phosphorylation sites were detected in our experiment, eight of which depicted decreased abundance-normalized phosphorylation, while 11 showed increased phosphorylation (Fig. 5C). Interestingly, sites showing decreased phosphorylation exhibited higher kinase library scores for MAPK1, MAPK3, and MAPK9 of the ERK and JNK signaling pathways (Fig. 5D–E; Fig. S3E), suggesting ERK and JNK kinases phosphorylate SINV proteins.

## DISCUSSION

Our study reveals a striking enrichment of human kinase motifs across viral proteomes, suggesting that viruses have evolved to biochemically sense host signaling pathways through post-translational modifications. By combining computational sequence-based kinase motif analysis, AI-based modeling of viral protein structures, and deep MS phosphoproteomics profiling of infected cells we found viral proteins harbor phosphorylated human kinase motifs predominantly recognized by stress, inflammation, and cell cycle kinases. Structural analysis further showed that these phosphorylation sites are often located on protein surfaces, where differences between human and viral proteins are most pronounced. Lastly, inhibiting MAPK signaling during Sindbis virus infection disrupted both viral replication and phosphorylation of key sites with high scoring kinase motifs for ERK and JNK kinases. Taken together, our results suggest that phosphorylation is not always a barrier for viruses to overcome, but a potential mechanism by which many viruses sense and respond to specific host cues, allowing them to adapt to the host intracellular environment.

One key class of signals viruses appear to monitor is the cellular stress response. Stress-activated kinases such as p38, JNK, and ERK are frequently activated in infected or damaged cells^23^, and our analysis identified an abundance of their target motifs within viral proteins. For viruses, sensing stress may provide a critical cue to shift from replication to assembly and egress, especially if the host cell is undergoing apoptosis or immune activation. A dying or distressed cell is a poor environment for completing a replication cycle, but may be optimal for producing and releasing progeny virions before cell death. Along these lines, herpesviruses are known to reactivate from latency in response to p38, ERK, and JNK kinase signaling, producing infectious virions to escape dying cells and infect neighboring healthy cells.^24^ Our findings suggest that many viruses may use these stress kinase signals as a form of temporal regulation to orchestrate their life cycle in synchrony with host cell viability.

In addition to stress, cell cycle kinases, such as cell cycle dependent kinases, also possess an abundance of high-confidence motifs in viral proteins. The cell cycle profoundly impacts the biochemical landscape of a cell, including the availability of dNTPs, nucleotide biosynthesis, chromatin accessibility, and replication machinery, all of which are resources that viruses exploit during their replication cycles.^25,26^ We found certain viral families to be enriched for cell cycle kinase motifs, including retroviruses, which are known to exploit the host nucleus for replication.^27^ The lentivirus subfamily of retroviruses integrate their genomes into the host genome, which requires coordination with host cell cycle activities and DNA replication machinery.^28^ Encoding motifs for cell cycle kinases may enable viruses to “read” the cell cycle state and tune their replication cycle to maximize fitness.

There are several limitations to our study. While our motif analysis and phosphoproteomic data suggest widespread kinase engagement across viral proteomes, definitive mechanistic studies will require precise mapping of phosphorylation site functionality on a case-by-case basis. Second, kinase motif prediction algorithms often fail to capture true in vivo substrate specificity, as they consider catalytic site affinity and neglect additional substrate features such as docking domains or scaffold interactions.^29^ Targeted perturbation studies will be needed to definitely link viral protein phosphorylation events to their upstream kinases in vivo.

Importantly, our findings open several translational avenues. For example, therapeutics could be developed to deliberately alter host signaling in ways that manipulate the viral life cycle, thereby reducing replication and giving the host immune system time to mount an effective response. Moreover, understanding how individual patient signaling responses affect viral processes could help to explain variability in disease severity and outcomes. Ultimately, this work lays the foundation for a deeper, systems-level understanding of how viruses exploit the dynamic cellular state to guide their life cycle and pathogenesis.

## METHODS

### COMPUTATIONAL METHODS

#### Curation of viral proteomes from Expasy

Viral protein sequences for 125 human-infecting viruses were collected from Expasy (virazone.expasy.org), where proteome files were linked to uniprot.org and downloaded as FASTA files. These sequences were processed in R to generate a data table linking each viral protein ID to its corresponding residue position and amino acid identity. This table was then used for downstream kinase motif analysis using the Kinase Library.

#### Curation of viral proteomes from Nomburg et al

Structural data in the form of PDB files for 21,606 viral proteins from 1,391 viruses were collected from publicly available Zenodo records.^20^ The PDB files were downloaded and stored locally. An R script was used to extract the RefSeq Protein ID from the file names, where it appears as an underscore-delimited string.

#### Kinase motif analysis

Fifteen-mer sequences centered on all serine, threonine, and tyrosine residues were generated from the human proteome, human-infecting viral proteomes, and the viral proteomes extracted from Nomburg et al. Each phosphoacceptor site was scored against the human kinase library, using serine/threonine kinases for S/T sites and tyrosine kinases for Y sites. Scores reflect predicted kinase and substrate compatibility, where 0 represents a random association and positive or negative values indicate favorable or unfavorable interactions. This analysis produced a table that assigns a kinase score to each phosphosite and kinase pair.

#### Calculation of relative surface accessibility (RSA) metric

Accessible surface area (ASA) for each residue was calculated using the Define Secondary Structure of Proteins (DSSP) tool on PDB files curated from Nomburg et al. and on structures generated independently with AlphaFold for HIV, KSHV, and SINV proteins. ASA values were then normalized by the maximum solvent-accessible surface area for each amino acid type, as reported by Tien et al. (2013), to calculate the relative surface accessibility (RSA) for each residue.

#### Defining high-confidence virus-kinase interactions

For each kinase, we compared viral kinase motif scores to human kinase motif scores for the same kinase across both the human-infecting viral proteome dataset and the dataset from Nomburg et al. To control for differences in sample size, human scores were downsampled to match the number of viral scores for each kinase. Analyses were only performed when at least 30 scores were available for both groups, in accordance with the assumptions of the Z-test. A Z-score was then calculated for each virus–kinase pair, representing the difference between viral and human kinase motif scores.

Significant virus–kinase interactions were defined as those with a Z-score greater than zero and a p-value less than 0.05. In addition, we required that each virus contain at least seven high-confidence sites for the kinase, where high confidence was defined as a motif score greater than two. This filtering process yielded a table of significant virus–kinase pairs for downstream analysis.

#### Gene set overrepresentation analysis of virus-associated kinases

Gene set overrepresentation analysis (GSOA) was performed using the curated list of significant virus–kinase interactions. For the human-infecting viral proteome dataset, we selected the top 50 kinases for each virus based on Z-score and performed GSOA using the Gene Ontology Biological Processes database. The background universe was defined as the HGNC names of all kinases included in our dataset. Pathway terms were ranked by the number of viruses associated with that term at an adjusted p-value below 0.05.

We also performed this analysis at the viral family level by selecting the top 50 kinases for each family, again ranked by Z-score, and applying the same GSOA approach. For the viral proteome dataset from Nomburg et al., we used Z-scores calculated for sites with RSA values greater than 0.25 and ranked kinases by their median Z-score across viruses. The top 37 kinases were selected for GSOA. Redundant pathway terms were removed from all figures for clarity.

#### Kinase motif comparison between virus and human

For human-infecting viruses, we selected phosphosite motifs that scored at least two for at least one kinase, defining these as high-confidence sites. The same filtering was applied to the human proteome. For each dataset, we calculated amino acid frequencies at each position within a ±7 residue window surrounding the phosphoacceptor site. These frequencies were then normalized by the background amino acid composition of the corresponding proteome. Finally, we computed the log2 ratio of the normalized viral frequencies to the normalized human frequencies at each position, generating an enrichment profile for amino acid usage surrounding high-confidence viral phosphosites.

#### AlphaFold2 analysis for HIV, KSHV, and SINV

Protein structures for the HIV, KSHV, and SINV proteomes were generated using ColabFold v1.5.5 with MMseqs2 Batch mode. FASTA sequences were input into ColabFold with five models and three recycles per prediction. The resulting PDB files were processed using DSSP to calculate accessible surface area (ASA) values for each residue, which were then used to compute relative surface accessibility (RSA).

#### Calculation of disorder metric

Intrinsically disordered regions were identified by calculating disorder scores for each residue using the IUPred2A algorithm, applied to a subset of proteins from the human proteome and the viral proteomes from Nomburg et al.^20^ These disorder scores were integrated with the relative surface accessibility (RSA) and kinase motif score metrics for downstream analysis.

#### In silico digestion of proteomes

We performed in silico digestion of the HIV, KSHV, SINV, and human proteomes using the cleaver package version 1.38.0, simulating trypsin digestion. Peptides were filtered to include fragments between seven and 52 amino acids in length. For downstream phosphoproteomics analyses, we used only tryptic peptides, as these are the fragments typically detectable by mass spectrometry.

#### Mass spectrometry proteomics data search and analysis

Mass spectra from each DIA dataset were searched against a database consisting of Uniprot Homo sapiens sequences downloaded on October 6, 2023, along with HIV, KSHV, and SINV proteomes. For protein abundance samples, data were searched using the default Biognosys settings, with methionine oxidation as a variable modification, carbamidomethylation of cysteine as a static modification, and a final false discovery rate of 1 percent applied at the peptide, peptide spectrum match, and protein levels. For phosphopeptide-enriched samples, Biognosys settings were modified to include phosphorylation of serine, threonine, and tyrosine as variable modifications. Phosphosite localization scores were applied using the PTM site localization feature in Spectronaut.

Quantitative analyses were performed in R. Initial quality control, including inter-run clustering, correlation analyses, principal component analysis, and assessment of peptide and protein counts and intensities, was conducted using the artMS package version 1.12.1. Statistical analysis of phosphorylation and protein abundance changes between mock- and virus-infected samples, as well as between infections from different viral variants, was performed using peptide ion fragment data from Spectronaut processed through artMS. Phosphorylation site quantification was conducted using artMS as a wrapper around MSstats, applying site conversion and quantification with default settings. Peptides containing the same set of phosphorylated sites were grouped and quantified as phosphorylation site groups.

For both phosphopeptide and protein abundance pipelines, MSstats performed normalization by median equalization without imputation of missing values. Median smoothing was applied to combine intensities from multiple peptide ions or fragments into a single intensity value for each protein or phosphorylation site group. Statistical testing for differences in intensity between infected and control samples was performed using the default MSstats settings. Unless otherwise specified, adjusted p-values were calculated using the Student’s t-test with the Benjamini–Hochberg method for false discovery rate correction, including cases where sample sizes were limited to two replicates.

#### Kinase activity analysis from phosphoproteomics data

We performed kinase activity analysis on phosphoproteomics data collected during HIV, KSHV, and SINV infection, as well as during a time course of SINV infection. Log2 fold changes in phosphosite intensities between infected and mock-treated cells were calculated and used to infer kinase activity states. Kinase–substrate relationships were determined using the Omnipath database, which provides curated kinase–substrate interaction scores.

### EXPERIMENTAL METHODS

#### Mass spectrometry proteomics sample processing

Samples were lysed in 6M guanidine hydrochloride (Sigma Aldrich), boiled at 95°C for 5 minutes, and stored on ice until sonication. Lysed samples were sonicated using a probe sonicator 2x for 15 seconds at 10% amplitude, and protein was quantified using a Bradford assay. Approximately 200 μg of protein sample was used for further processing, starting with reduction and alkylation using a 1:10 sample volume of tris-(2-carboxyethyl) (TCEP) (10 mM final) and 2-chloroacetamide (40 mM final) for 5 minutes at 45°C with shaking at 1500 rpm. Before protein digestion, the 6 M guanidine hydrochloride was diluted 6-fold with 100 mM Tris-HCl (pH 8) to permit the activity of the proteolytic enzyme trypsin. Trypsin was added at a 1:100 (wt/wt) enzyme-substrate ratio and placed in a thermomixer at 37°C overnight (16 h) with shaking at 800 rpm. Following digestion, 10% trifluoroacetic acid (TFA) was added to each sample to a final pH of approximately 2. Samples were then desalted using a vacuum manifold with 50 mg Sep Pak C18 cartridges (Waters). Each cartridge was activated with 1 mL of 80% acetonitrile (ACN)/0.1% TFA, then equilibrated with 3 × 1 mL of 0.1% TFA. After sample loading, cartridges were washed with 3 × 1 mL of 0.1% TFA, and samples were eluted with 1 × 0.8 mL 50%ACN/0.25% formic acid (FA). Approximately 10% of the desalted peptides were separated for global proteomics analysis and the remaining 90% were used for phosphopeptide enrichment. Both the fractions were then dried by vacuum centrifugation. The Ti-IMAC HP beads (Resyn Biosciences) were used according to the manufacturer’s instructions for phosphopeptide enrichment. After the elution of phosphopeptides from the beads, the pH was brought down immediately to pH of approximately 3 with FA (10% (v/v) in LC/MS grade water). Samples were dried by vacuum centrifugation and stored at −80°C until further analysis.

#### Mass spectrometry proteomics acquisition

Dried peptides were resuspended in 0.1% (v/v) FA in MS grade water and analyzed on a timsTOF HT mass spectrometer, paired with a Vanquish Neo UHPLC system. Mobile phase A consisted of 0.1% (v/v) FA in MS grade water, and mobile phase B consisted of 0.1% (v/v) FA in 100% MS grade Acetonitrile. The LC was operated in trap-and-elute mode, where the peptides were first trapped onto a PepMap Neo Trap column (5 mm, 100 Å pore size, 5 μm particle size) and then reversed-phase separated using gradients mentioned below on an Aurora Elite C18 reverse phase column (15 cm, 100 Å pore size, 1.5 μm particle size for captive spray, IonOptiks), kept at 50°C using a column oven for Bruker Captive Spray source (Sonation Lab Solutions), and ionized in a CaptiveSpray source (Bruker Daltonics) at 1700 V. For BG2 phosphoproteome analysis trapped peptides were separated at 400 nL/min flow rate, on a gradient of %B as follows: 3% to 4.5% B over 4 min at 1 μL/min, then to 5% in next 0.5 min, and 28% B in 31.5 min, followed by an increase to 45% B over 3 min, and to 60% B over 1 min. For BG2 global proteome analysis, the %B gradient used was: 5% to 8% over 4 min at 1μL/min, then to 10% in the next 1 min, and 28% in the next 28 min, followed by an increase to 45% B over 6 min, and 60% B over 1 min at 400 nL/min flow rate. For BG4 global abundance analysis, the LC gradient was set as follows: 5% to 8% B over 1 min, then to 30% B in 36 min, followed by an increase to 45% B over 5 min at 300 nL/min flow rate. For BG4 phosphoproteome analysis, the %B gradient used was 3% to 16% over 25 min, 30% in the next 12 min, 45% in the next 4 min, and 60% B over 1 min at 300 nL/min flow rate.

For phosphoproteome analysis, the raw data was acquired in dia-PASEF mode with variable isolation window widths in the m/z vs ion mobility plane. These windows were adjusted to maximize the coverage of precursor ions. For dia-PASEF, in the ion mobility (1/K0) range 0.6 to 1.50 Vs cm-2, the collision energy was linearly decreased from 59 eV at 1/K0 = 1.6 Vs cm-2 to 20 eV at 1/K0 = 0.6 Vs cm-2 to collect the MS/MS spectra in the mass range 400.2 to 1399.3 Da. The estimated mean cycle time was 1.38 s. For the BG2 global abundance data analysis, equal-size windows of 28 Da were designed with an overlap of 1 Da to maximize the precursors ion coverage for further MS/MS. The ion accumulation time and ramp times in the dual TIMS analyzer were set to 100 ms each. In the ion mobility (1/K0) range 0.6 to 1.47 Vs cm-2, the collision energy was linearly decreased from 59 eV at 1/K0 = 1.47 Vs cm-2 to 20 eV at 1/K0 = 0.6 Vs cm-2 to collect the MS/MS spectra in the mass range 193.1 to 1328.1 Da. For BG4 global abundance analysis, the collision energy was linearly decreased from 59 eV at 1/K0 = 1.39 Vs cm-2 to 20 eV at 1/K0 = 0.67 Vs cm-2 to collect the MS/MS spectra in the mass range 267.7 to 1300.7 Da. The estimated mean cycle time was 1.59 s.

#### Mass spectrometry phosphoproteomics in cells with reactivated HIV

The J-Lat 10.6 cells were seeded and maintained up to a week in RPMI-1640 medium (Gibco) supplemented with 10% fetal bovine serum (FBS, Gibco) and 1% penicillin/streptomycin (Gibco) at 37 °C and 5% CO_2_. To check the effect of phorbol 12-myristate 13-acetate (PMA) on HIV-1 reactivation, 10 million J-Lat 10.6 cells were treated in a 10 cm cell culture dish with PMA at a final concentration of 5 ng/mL and harvested after 24 h. The collected cells were spun at 400 g for 5 min at 4 °C to remove the cell culture media, and the pellet was washed two times with ice-cold 1× PBS (Corning).

Next, cells were lysed by adding 300 μL of lysis buffer (4% sodium deoxycholate, 0.1 M Tris-HCl, pH 8) and sonication using a tip sonicator (Fisher Scientific). The samples were then boiled at 95 °C for 5 min and centrifuged at 18,000 g for 10 min at 4 °C to clear out cell debris. The supernatant was transferred to a new protein LoBind tube (Eppendorf) and subjected to protein concentration estimation using the BCA assay kit (Pierce). Volumes corresponding to 1 mg of proteins were taken from each sample and normalized to 200 μL with the lysis buffer.

Proteins were reduced with 10 mM TCEP (tris(2-carboxyethyl)phosphine, Pierce) at 56 °C for 30 min and alkylated with 40 mM 2-chloroacetamide (Thermo Scientific) at 23 °C in the dark for 30 min on a ThermoMixer (Eppendorf) at 800 rpm. Proteins were cleaned up with Sera-Mag SpeedBeads Carboxyl magnetic beads (Cytiva). A 1:1 (w/w) mixture of hydrophilic and hydrophobic beads was prepared. The volume of beads corresponding to a 1:10 (w/w) protein:beads ratio was aliquoted and washed three times with 1 mL of LC-MS grade water (Fisher Scientific).

Proteins were added to the washed beads and mixed at 23 °C for 5 min at 1200 rpm. Protein binding was performed by adding ethanol (Fisher Scientific, molecular biology grade, final concentration 50% v/v) and shaking at 1200 rpm for 15 min. The unbound fraction was discarded, and the beads were washed three times with 1 mL of 80% ethanol (v/v) in LC-MS grade water. The beads were then resuspended in 0.1 M ammonium bicarbonate buffer (pH 8) and digested overnight at 37 °C, 1200 rpm, using a protein:protease ratio of 1:100 (w/w) with Trypsin (Promega) and Lys-C (Wako).

After digestion, the supernatant was transferred to a new protein LoBind tube (Eppendorf), and the beads were washed twice with 0.1 M ammonium bicarbonate buffer (pH 8). The eluates were pooled. Trifluoroacetic acid (TFA) was added to a final 0.1% (v/v) concentration. Five percent of the eluted peptides were separated for global proteomic analysis, and the remainder was used for phosphopeptide enrichment. Both fractions were dried to completeness in a SpeedVac (Labconco).

For phosphopeptide enrichment, Ti-IMAC beads were aliquoted in a 1:2 (w/w) peptide:beads ratio and equilibrated three times with binding buffer (0.1 M glycolic acid in 80% acetonitrile (ACN), 5% TFA). Dried peptide samples were resuspended in 200 μL of binding buffer, added to the equilibrated beads, and incubated for 30 min at 23 °C, 1200 rpm. The unbound fraction was discarded, and beads were washed sequentially once each with 200 μL of the binding buffer, wash buffer 1 (60% ACN, 1% TFA, 200 mM NaCl), wash buffer 2 (60% ACN, 1% TFA), and finally with LC-MS grade water.

Enriched phosphopeptides were eluted twice by incubating the beads with 150 μL of 1% (v/v) ammonium hydroxide (Sigma) in LC-MS grade water (Fisher Scientific) for 10 min at 23 °C, 1200 rpm. The eluted peptides were transferred to a new protein LoBind tube (Eppendorf) containing 50 μL of 10% (v/v) formic acid in LC-MS grade water. Both eluates were pooled and dried in a SpeedVac (Labconco). Dried peptides were resuspended in 0.1% formic acid, and a volume corresponding to 500 ng of peptides was analyzed using timsTOF HT (Bruker Daltonics).

#### SINV time course experiment

A total of 3 × 10^6^ HEK293T cells were seeded per 10 cm dish. The following day, cells were either mock-infected or infected with EGFP-labeled Sindbis virus (SINV) strain TE5’2J/GFP at a multiplicity of infection (MOI) of 0.01. Infections were carried out in 2 mL of 1% FBS in PBS at 37°C for 1 hr to allow virus adsorption. After adsorption, 8 mL of complete growth medium (DMEM supplemented with 10% FBS and 1% penicillin-streptomycin) was added to each plate to mark the 0 hr time point. Cells were then incubated at 37°C for the indicated durations prior to lysis. At the 16 hr time point, cells were treated with either ruxolitinib (1 uM, MedChemExpress) or the MAPi inhibitor cocktail consisting of doramapimod (1 uM, MedChemExpress), JNK-IN-8 (1 uM, Selleckchem), and U-0126 (1 uM, Selleckchem).

#### SINV luciferase experiment

A total of 1 × 10^6^ HEK293T cells were seeded per well in 24-well plates. The following day, the culture medium was aspirated, and the cells were treated with either MAPi or ruxolitinib (1 uM) in 150 uL 1% FBS in PBS. After 1 hr incubation with the inhibitors at 37C, SINV (add strain info, the luciferase one), was added directly to the inoculum at an MOI of 1. Following a 1hr virus adsorption period at 37C, 350uL of complete growth medium was added to each well to mark the 0-hr time point. After 24 hr infection, luminescence was recorded using a plate reader following the manufacturer’s protocol for the Promega Luciferase Assay System (Promega).

#### MTT assay

At the indicated time point, the culture medium was replaced with 500 uL of fresh complete medium, and 10 uL of 12mM MTT stock solution (prepared in PBS) was added to each well. Plates were incubated for 2hr at 37 C. 500 uL of SDS-HCl (10% SDS in 0.01M HCl), was added to each well and the plates were incubated for another 4hr. Wells were mixed by pipetting, and the absorbance was measured at 570 nm using a microplate reader. To calculate cell viability, background absorbance from wells containing media and reagents with no cells was subtracted from all measurements. Absorbance values for each condition were then normalized to the average absorbance of untreated control wells to calculate the percentage of viable cells.

#### Cell lysis and digestion

Cells were lysed in a denaturing buffer consisting of 8M urea, 0.1M Tris-HCl, 250 mM NaCl, and 50mM ammonium bicarbonate, supplemented with protease (mini-cOmplete, Roche) and phosphatase inhibitors (PhosSTOP, Roche). Lysates were immediately frozen at −80°C. Upon thawing, DNA was sheared via probe sonicated on ice at 15% amplitude for 15 s per cycle, with 2 cycles performed per sample.

Protein concentrations were determined using the Bradford assay. For each sample, approximately 1 mg of total protein was processed. Disulfide bonds were reduced with 10 mM tris(2-carboxyethyl)phosphone (TCEP) for 30 mins at 25°C with shaking (1500 rpm), followed by alkylation with 40 mM 2-chloroacetamide (2-CAM) under the same conditions. Samples were then diluted from 8M to 2M urea with 0.1M Tris-HCl to facilitate protease activity. Proteins were digested overnight (18 hr) at 25°C with shaking (800 rpm) using sequencing-grade trypsin (Promega) at a 1:100 (wt/wt) enzyme-to-substrate ratio, and Lysyl Endopeptidase (Lys-C, Irvine Scientific), at a 1:50 (wt/wt) ratio.

Following digestion, samples were acidified with 10% trifluoroacetic acid (TFA) to achieve a final pH of 2–3. Acidified samples were clarified by centrifugation at 18,000 xg for 10 mins at 4°C. Peptides were desalted with Oasis HLB cartridges (waters) on a vacuum manifold. The cartridges were first activated with 1 mL 80% acetonitrile (ACN)/0.1% TFA. Columns were then equilibrated with 1mL 0.1% TFA 3 times. Samples were loaded twice onto the cartridges to ensure maximal binding. Bound peptides were then washed 3 times with 1mL 0.1% TFA. Peptides were eluted with 800 uL of 50% ACN/0.25% formic acid (FA). Approximately 10% of each sample was kept for protein abundance measurements, and the remainder was used for phosphopeptide enrichment. Samples were dried by vacuum centrifugation and stored at −80°C until further analysis.

#### Phosphopeptide enrichment

Phosphorylated peptides were enriched using Ti-iMAC HP magnetic beads (ReSyn Biosciences), at a peptide-to-bead ratio of 1:2 according to the manufacturer’s protocol. After elution of phosphopeptides from the magnetic microparticles, the pH was immediately brought down to pH = 3 with 10% FA. Samples were dried by vacuum centrifugation and stored at −80°C until further analysis.

## Supplementary Material

Supplement 1

Supplement 2

Supplement 3

Supplement 4

Supplement 5

Supplement 6
