# Supplementary material for: Global Landscape of Human Kinase Motifs in Viral Proteomes
Source: bioRxiv. 2025 Jun 3:2025.06.02.657064. Preprint. [Version 1] doi: 10.1101/2025.06.02.657064 (PMC12157635; doi:10.1101/2025.06.02.657064)
Supplement: Supplement 6 [file NIHPP2025.06.02.657064v1-supplement-6.pdf]

bioRxiv preprint doi: https://doi.org/10.1101/2025.06.02.657064; this version posted June 3, 2025. The copyright holder for this preprint (which was not certified by peer review) is the author/funder, who has granted bioRxiv a license to display the preprint in perpetuity. It is made available under aCC-BY 4.0 International license.

SUPPLEMENTAL FIGURES

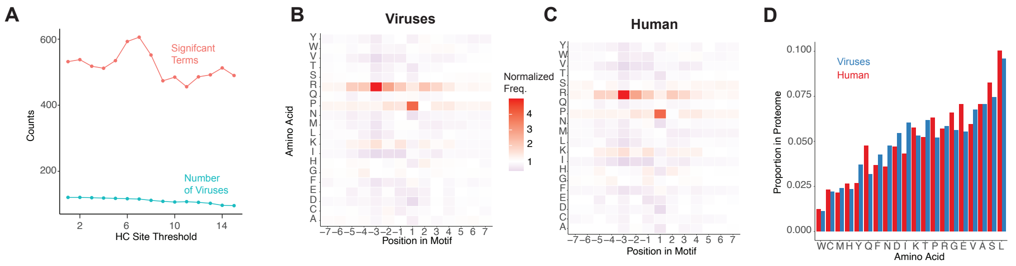

Supplemental Figure 1. Identification of significant virus-kinase pairs and proteome composition of viruses and humans. (A) Performance of different threshold values for defining high-confidence sites per virus-kinase pair. Red indicates the number of significantly enriched GSOA terms ($p < 0.05$), while blue shows the number of viruses retained in the GSOA analysis after applying each threshold. (B) Amino acid distribution within high-confidence viral kinase motifs, normalized to the background distribution across viral proteomes. (C) Amino acid distribution within high-confidence human kinase motifs, normalized to the background distribution across human proteomes. (D) Baseline amino acid composition across viral (blue) and human (red) proteomes.

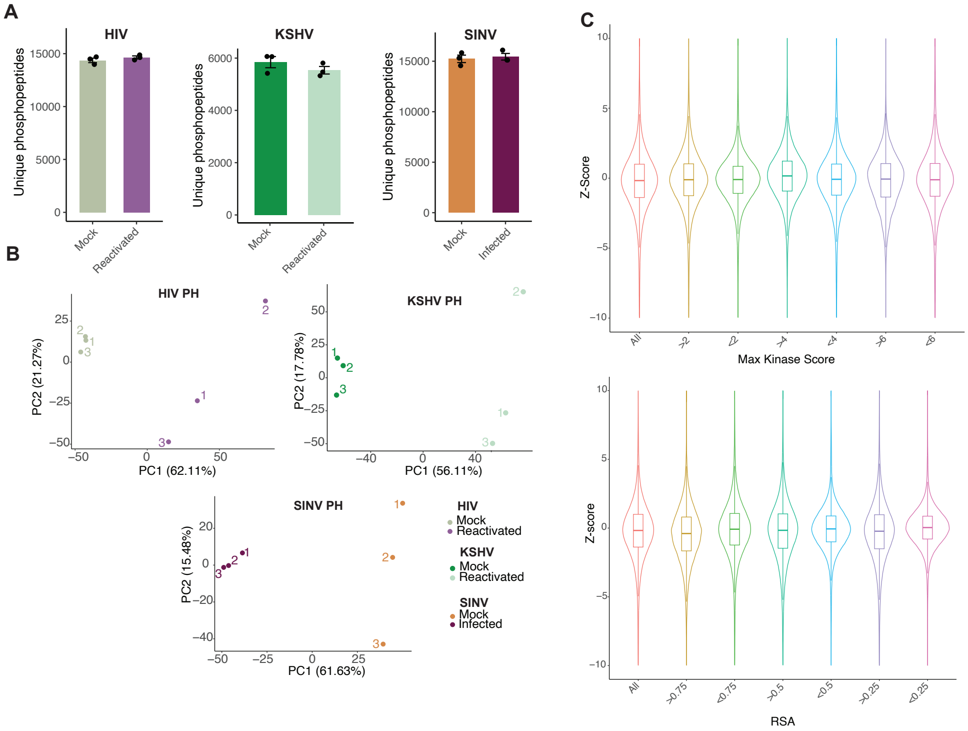

Supplemental Figure 2. Quality control of phosphoproteomic samples and filtering threshold determination. (A) Number of unique phosphopeptides detected across three biological replicates for SINV infection in HEK293T cells, KSHV reactivation in B cells, and HIV reactivation in J-Lat 10.6 cells. (B) Principal component analysis (PCA) of phosphoproteomics data for SINV infection, and KSHV and HIV reactivation. (C) Comparison of different filtering thresholds using Z-score analyses for virus–human kinase comparisons. RSA and maximum kinase score were tested as filtering metrics with three different thresholds. Z-scores were computed per

bioRxiv preprint doi: https://doi.org/10.1101/2025.06.02.657064; this version posted June 3, 2025. The copyright holder for this preprint (which was not certified by peer review) is the author/funder, who has granted bioRxiv a license to display the preprint in perpetuity. It is made available under aCC-BY 4.0 International license.

virus–kinase pair.

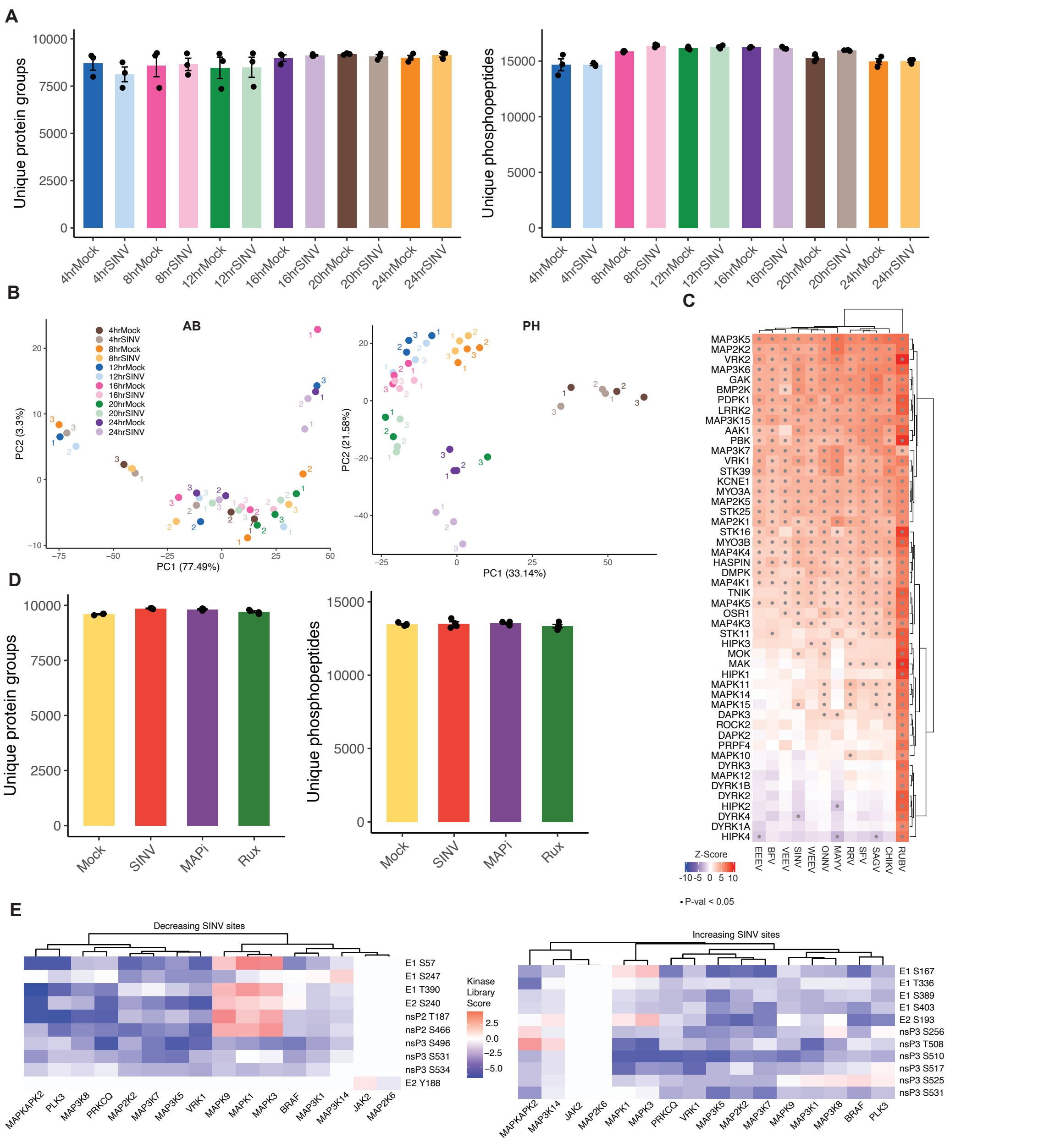

Supplemental Figure 3. Sindbis virus time-course and inhibitor experimental quality control. (A) Number of unique protein groups (left) and unique phosphopeptides (right) across different conditions in the SINV time-course infection experiment. Each dot represents a biological replicate. (B) Principal component analysis (PCA) of total protein abundance data (left) and phosphoproteomics data (right) from the SINV time-course infection. (C) Z-scores for Togaviruses across various kinases within the stress-activated kinase family. (D) Number of unique protein groups (left) and unique phosphopeptides (right) across conditions in the SINV inhibitor experiment. Each

bioRxiv preprint doi: https://doi.org/10.1101/2025.06.02.657064; this version posted June 3, 2025. The copyright holder for this preprint (which was not certified by peer review) is the author/funder, who has granted bioRxiv a license to display the preprint in perpetuity. It is made available under aCC-BY 4.0 International license.

dot represents a biological replicate. (E) Kinase library scores for MAPK kinases corresponding to phosphosites that decrease (left) or increase (right) upon MAPK inhibitor treatment.

SUPPLEMENTARY TABLES

Table S1. Metadata for Human-Infecting Viruses
Includes viral taxonomic classification, proteome composition, number of high-confidence kinase motifs, total STY sites, proteome size, and proportion of high-confidence motifs for all kinase families.

Table S2. Z-Scores for Virus–Kinase Motif Enrichment
Z-scores, p-values, and high-confidence motif counts for each virus–kinase pair, including significance marking based on applied thresholds.

Table S3. GSOA Enrichment Results
Sheet 1: GSOA enrichment for human-infecting viruses.
Sheet 2: GSOA enrichment for human-infecting viruses, grouped by viral families.
Sheet 3: GSOA enrichment for the Nomburg et al. dataset.

Table S4. Viral Protein Abundance and Phosphorylation in SINV Experiments
Sheet 1: Viral protein and phosphorylation intensities over time during SINV infection.
Sheet 2: Viral protein and phosphorylation levels in the inhibitor experiment.

Table S5. Kinase Activity During SINV Time Course
Calculated kinase activities based on phosphoproteomics data from the SINV time course.
